# Assessment of the Effect of Erenumab on Efficacy and Quality-of-Life Parameters in a Cohort of Migraine Patients With Treatment Failure in Cyprus

**DOI:** 10.3389/fneur.2021.687697

**Published:** 2021-07-29

**Authors:** Andria Tziakouri, Haritini Tsangari, Costas Michaelides

**Affiliations:** ^1^Department of Neurology, American Medical Center, Nicosia, Cyprus; ^2^Department of Accounting, Economics and Finance, School of Business, University of Nicosia, Nicosia, Cyprus; ^3^Department of Neurology, Medical School, University of Nicosia, Nicosia, Cyprus

**Keywords:** erenumab, chronic migraine, quality of life, MSQ V2.1, Cyprus, treatment failure

## Abstract

Erenumab is the first human monoclonal antibody to be approved as a selective therapy for migraine prophylaxis in adults. This study assessed, in a real-world setting, the efficacy of erenumab and its impact on the quality of life (QoL) of Cypriot migraine patients who had failed several treatments in the past. Erenumab was prescribed as a stand-alone or as an add-on therapy to 16 patients with chronic migraine. The first component of the study examined migraine parameters before and after erenumab therapy and included an interim 3-month subjective assessment. In the second component, the patients were asked to complete the validated Migraine-Specific Quality-of-Life Questionnaire—Version 2.1 (MSQ V2.1) during the last month of their individual treatment as a measure of the QoL. The results showed a statistically significant improvement in almost all migraine parameters following erenumab treatment. In the 3-month-interval assessment, 81.3% of the patients reported an improvement in their mental well-being, anxiety, and depression levels, with more than 80% of the patients reporting an improvement in almost all assessed migraine parameters. MSQ V2.1 indicated a good health status in all three domains (mean values > 60 on a scale 0–100), with the “role function preventative” domain having the highest health scores (85). Over a period of 6 months, erenumab was safe, well-tolerated, and effective in preventing migraine symptoms and improving HR-QoL. We conclude that this novel medication, which is not yet part of the national formulary in Cyprus, may be a cost-effective solution in reducing the disease burden of chronic migraine.

## Introduction

Migraine is one of the four most common causes of disability worldwide according to the World Health Organization (1, 2). Up until recently, the available treatments for migraine have included non-specific oral prophylactic medications such as anti-epileptic drugs, tricyclic antidepressants (TCAs), beta-blockers, calcium channel blockers, and selective serotonin reuptake inhibitors (SSRIs) among others as well as injectable therapies, like botulinum toxin, that were approved for the treatment of other conditions and were later repurposed as migraine treatments (3). They are used alone or in combination, with variable efficacy and tolerability often leading to decreased compliance, frequent medication switching, and overuse of acute migraine medication (4, 5).

The Republic of Cyprus has a population of <1 million people, with a percentage of migraine patients comparable to that of other developed countries (nearly 10%) (6). The traditional migraine-preventative medications used worldwide are also available in Cyprus, with only two migraine-specific rescue treatments available (zolmitriptan and sumatriptan), both in *per os* formulation. Over-the-counter medications include codeine-containing formulations and contribute to the high prevalence of medication-overuse headache (MOH; defined as the use of pain relief medications alone or in any combination for at least 10 days per month or more) (7).

Erenumab, a calcitonin gene-related peptide (CGRP) receptor monoclonal antibody, has been developed as a selective therapy for the treatment of acute and chronic migraine in adults (8–11). The once-monthly self-injectable drug received approval by the European Medicines Agency in July 2018 (12). Erenumab has been available commercially to Cypriot patients since February 2019. Despite the recent introduction of a new healthcare system in the Republic of Cyprus, erenumab is currently not offered through this scheme, forcing the majority of the patients to pay out of their pocket to purchase it, with only a minority getting it through private health insurance coverage.

Although real-life data from empirical studies confirm the effectiveness and tolerability of erenumab in the treatment of both episodic and chronic migraine, including therapy-refractory populations (9–11, 13–16), its high cost for the patients in Cyprus makes its use very challenging. The goal of this study was to assess, in a real-world setting, the efficacy of erenumab and its impact on the quality of life (QoL) of Cypriot migraine patients with treatment failure.

## Methods

### Study Design and Participants

A prospective observational cohort study was conducted. Patients were eligible if they suffered from chronic migraine which, according to the International Classification of Headache Disorders (17), is defined as 15 or more monthly migraine days for more than 3 months which, on at least 8 days/month, has the features of migraine headache and if they had failed treatment with at least three preventative medications. The recruitment period was 6 months, and during this period, 16 patients satisfied the eligibility criteria, as determined by their treating neurologists, and were thus recruited for the study. Each patient obtained the medication either by paying out of their pocket or after approval by their private health care insurance provider (if available). The patients were allowed to continue taking other preventative oral or injectable therapies, and the minimum duration of participation in the study was 6 months. All patients provided their written informed consent to participate in the study. The protocol was reviewed and approved by the National Committee of Bioethics of Cyprus (EEBK EM 2020.01.58).

The study included two components. The first component of the study examined migraine parameters (shown on **Table 3**) before erenumab therapy and during the last month of individual treatment for each patient. The data were collected by the study investigator who interviewed the patients at these two points in time, examining the monthly migraine frequency (including the number of missed and limited days defined as the necessity of bed rest or isolation and the decrease in the daily productivity of the patient, respectively), the subjective severity (1–10), the associated migraine symptoms, and the monthly use of acute medication.

The first component also included an interim 3-month subjective assessment which evaluated the qualitative effect of erenumab, using a “yes or no” questionnaire prepared by the study investigators, examining the perceived change of different migraine parameters as well as the subjective improvement of depression levels, anxiety levels, and physical and mental well-being after three doses.

### Migraine-Specific Quality-of-Life Questionnaire—Version 2.1

In the second component, the patients were asked to complete the validated Migraine-Specific Quality-of-Life Questionnaire—Version 2.1 (MSQ V2.1) during the last month of their individual treatment as a measure of QoL. The patients had the option to answer the questionnaire in either English or Greek language (permission for translation was given by GlaxoSmithKline, Inc., the owner of MSQ V2.1 copyright; distributed by Mapi Research Trust)^1^. The survey instrument, MSQ V2.1, is a 14-item questionnaire that evaluates the impact of migraine on health-related quality of life (HR-QoL) over a period of 4 weeks, assessing three different dimensions: role function—restrictive (RR), role function—preventive (RP), and emotional function (EF) (18).

The RR domain comprises seven items and evaluates the difficulty in performing daily activities due to migraine symptoms, the RP domain comprises four items and measures the extent to which daily activities are completely interrupted, and EF comprises three items assessing the effects of migraine on the emotional state of the patient regarding his or her feelings as being a burden to others (19). All items are measured on a six-point Likert scale, from 1 (never) to 6 (all the time). The validity and reliability of the MSQ have been shown in several studies (20–22).

### Study Outcomes

The primary endpoint of this study was patient HR-QoL and included the MSQ scores at the end of the individual treatment with erenumab for each patient and a 3-month subjective interim analysis assessing a change in migraine parameters and anxiety and depression levels as well as mental and physical well-being after 3 months of erenumab treatment. Secondary endpoints evaluated the efficacy of erenumab and included the change from baseline in monthly migraine days (MMDs; including change in missed and limited days), monthly acute medication days (AMDs), pain intensity, and migraine-associated symptoms.

The following secondary endpoints were also assessed: percentage of reversion from chronic migraine to episodic migraine (i.e., patients who changed from ≥15 migraine days per month to ≤ 14 migraine days per month) and percentage of patients converting from medication overuse headache to non-medication overuse headache after at least 6 months of erenumab therapy.

### Statistical Analysis

Descriptive statistics were obtained for the socio-demographics and clinical characteristics of the study participants (mean/standard deviation for numerical variables and frequency/percentage for categorical variables).

For each migraine parameter, the mean, standard deviation (SD), and median were calculated. Normality tests (Shapiro–Wilk) first examined if the variables were normally distributed, and then, accordingly, parametric or non-parametric tests were implemented to examine whether the improvement in each migraine parameter was statistically significant: paired-samples *t*-test for the normal variables and Wilcoxon signed-rank test for the non-normal variables.

For the assessment of the MSQ V2.1, since the items are worded with a negative perspective, they were first recoded before the domain scores were calculated. The computation of the raw domain scores was done, and then the transformation of the raw domain scores to a 0–100 scale was performed based on the scoring instructions. The transformation process allows each domain to reflect the percentage of the total possible score achieved (since 100 equals the highest score, thus a higher score indicates better health status). A transformation procedure was similarly done for the total scale. Reliability analysis included Cronbach's alpha as well as calculation of “alpha if item deleted.” Values of alpha close to 1 show a high internal consistency (23).

All the analyses were performed with the statistical software SPSS, version 25.0.

## Results

### Socio-Demographics and Patient Clinical Characteristics

The socio-demographic and clinical characteristics of the patients are shown in Table 1. Erenumab was prescribed as a stand-alone therapy for four patients (25.0%) and as an add-on therapy for 12 patients (75%): seven patients (43.8%) were on one additional preventative medication, while five patients (31.3%) were on two concurrent preventative medications at the onset of erenumab treatment. All 16 patients (100%) reported using triptans at some point in their life as an acute treatment for their migraine.

**Table 1:** Socio-demographics and clinical characteristics of the cohort sample.

	**All patients** **(*N* = 16)**
Age, years mean (SD)	43.8 (8.7)
**Sex**	
• Female, *n* (%)	14 (87.5)
• Male, *n* (%)	2 (12.5)
**Working status**	
• Employed, *n* (%)	13 (81.2)
• Retired, *n* (%)	1 (6.3)
• Unemployed, *n* (%)	2 (12.5)
**Relationship status**	
• Single, *n* (%)	3 (18.8)
• In a relationship, *n* (%)	2 (12.5)
• Married, *n* (%)	11 (68.7)
**Living situation**	
• Living independently in a household (with spouse or significant other)	13 (81.2)
• Living in residence with a family member (not spouse or significant other)	3 (18.8)
**Highest level of education**	
• Secondary school, *n* (%)	1 (6.3)
• University, *n* (%)	15 (93.7)
Smoker, *n* (%)	1 (6.3)
Family history of migraine, *n* (%)	7 (43.8)
**Referral to a neurologist**, ***n*** **(%)**	
• Alone	6 (37.5)
• GP	2 (12.5)
• Other	8 (50.0)
**Chronic concurrent preventative medication with erenumab**, ***n*** **(%)**	
• None	4 (25)
• Erenumab as second line of treatment	7 (43.8)
• Erenumab as third line of treatment	5 (31.3)
**Use of triptans**, ***n*** **(%)**	
• At some point in their life	16 (100.0)
• At onset of erenumab treatment	9 (56.3)
• Stopped during erenumab treatment	1 (6.3)
**Patients fulfilling the criteria of medication-overuse headache (MOH) (%)**	14 (87.5)
• Conversion of MOH after at least 6 months of erenumab treatment	8 (57.1)

Thirteen patients were prescribed with the 70-mg erenumab formulation for the first 2 months and were titrated up to the 140-mg formulation for the remainder of the treatment, while three patients were started directly on the 140-mg formulation as per the decision of the treating neurologist. Two patients had discontinued the treatment after 3 months; one was unable to continue paying for the medication, and the other withdrew due to low efficacy. Both patients were included in the statistical analysis.

### Primary Endpoints

#### Migraine-Specific Quality-of-Life Questionnaire—Version 2.1

All patients, except three, fully completed the MSQ V.2.1 on the last month of their individual treatment (two patients had withdrawn from the study after 3 months due to financial reasons and low effectiveness, respectively, and one patient refused to answer the questionnaire). Thus, based on the scoring instructions, no analysis or estimation of the missing data was performed.

Cronbach's alpha values were found to be very high (close to 1), both for the total MSQ scale and the three domains, while the alpha values did not improve significantly if any item was deleted. These results show the high internal consistency and reliability of the MSQ V2.1 instrument in the present study.

As seen in Table 2, HR-QoL assessed by the MSQ V2.1 during the last 4 weeks of erenumab therapy indicated a better health status in all three domains of the questionnaire (with mean values being higher than 60). The domain that had the highest health scores was “role function—preventive,” with 50% of the values being over 85 and with a mean of 75.4.

**Table 2:** Results obtained by the migraine-specific quality-of-life (MSQ) questionnaire (assessed during the last 4 weeks of erenumab treatment).

**Domains (MSQ items)**	**Number of** **items**	**Cronbach's** **alpha**	**Raw domain scores, range**	**Transformed scores, range** **(0–100 scale)**	**Mean (SD) of transformed scores** **(0–100 scale)**	**Median of transformed series**
1. Role function restrictive (1–7)	7	0.953	12–40 (possible: 7–42)	14.3–94.3	62.2 (21.3)	60.0
2. Role function preventive (8–11)	4	0.811	11–24 (possible: 4–24)	35.0–100.0	75.4 (18.9)	85.0
3. Emotional function (12–14)	3	0.910	7–18 (possible: 3–18)	26.7–100.0	65.1 (28.6)	60.0
Total MSQ scale	14	0.961	31–79 (possible: 14–84)	24.3–92.9	66.6 (20.8)	64.3

#### 3-Month-Interval Assessment of Erenumab Therapy

**Figure 1:**
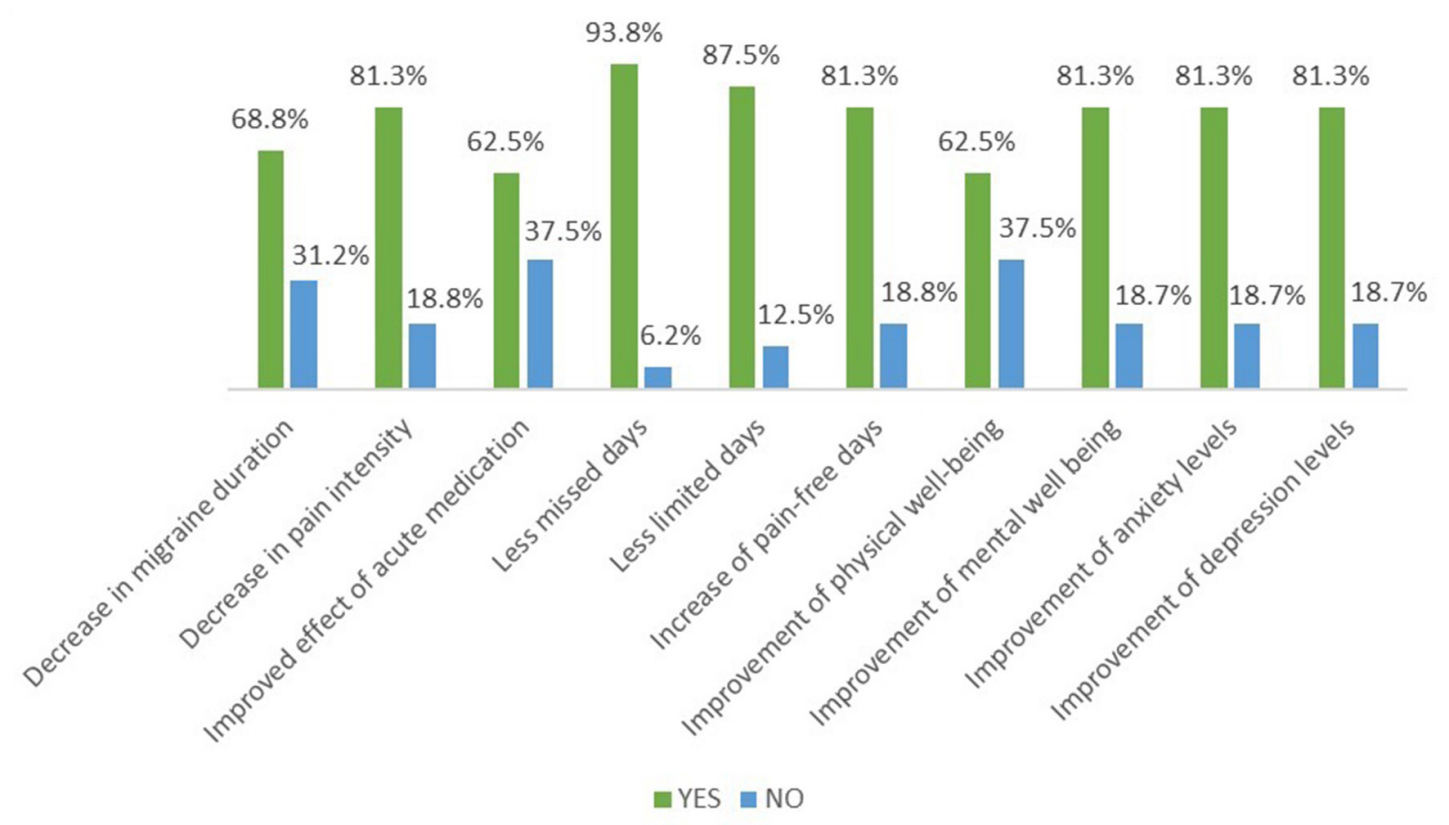
Three-month interval subjective assessment of erenumab therapy. The vast majority of the patients reported an improvement of all the parameters shown.

At this study interval, 68.8% of patients reported a decrease in migraine duration, while 81.3% experienced a decrease in pain intensity and 62.5% reported an improved effect of acute medication (Figure 1). Subjective improvement of both missed and limited days in a month was noted by 93.8 and 87.5% of patients, respectively, along with 81.3% of patients reporting an increase of monthly pain-free days. Self-reported improvement of physical well-being was seen in considerably more than half of the patients (62.5%), while 81.3% of participants noted a positive change on their overall mood, including improvement of mental well-being, anxiety levels, and depression levels, all in the first 3 months of treatment. All (100%) the patients would recommend erenumab to another patient after the 3 first months of treatment.

### Secondary Endpoints

#### Migraine Parameters Before and After Erenumab Treatment Initiation

Table 3 compares the eight migraine parameters before and after erenumab therapy (a figure has also been included as supplementary material in Appendix). The paired-samples *t*-test was performed for parameters 1, 5, and 8 since a statistical examination with Shapiro–Wilk showed that these variables were normally distributed both before and after therapy (parameter 1: *p* = 0.760 before, *p* = 0.304 after; parameter 5: *p* = 0.339 before, *p* = 0.079 after; and parameter 8: *p* = 0.387 before, *p* = 0.199 after). The Wilcoxon signed-rank test was used for testing if significant differences existed in the other variables which were non-normal (parameter 2: *p* = 0.073 before, *p* = 0.001 after; parameter 3: *p* = 0.175 before, *p* = 0.007 after; parameter 4: *p* = 0.015 before, *p* = 0.307 after; parameter 6: *p* = 0.642 before, *p* = 0.017 after; and parameter 7: *p* = 0.002 before, *p* = 0.017 after). As shown from the mean and median values, all migraine parameters improved following erenumab treatment, and the change was highly significant for almost all migraine parameters (*p* < 0.001 for parameters 1, 5, and 8; *p* = 0.001 for parameters 2 and 4; and *p* = 0.003 for parameter 6). Only two parameters did not have a statistically significant improvement, namely, “number of limited days per month” (*p* = 0.426) and “number of migraine attacks free of accompanying symptoms per month” (*p* = 0.138).

**Table 3:** Migraine parameters before and after erenumab therapy.

	**Before erenumab** **therapy initiation**		**During/after erenumab** **therapy initiation**		
**Migraine parameters**	**Mean ± SD**	**Median**	**Mean ± SD**	**Median**	***p*-value**
1. Number of monthly migraine days	22.00 ± 6.75	21.00	13.00 ± 8.69	11.50	<0.001^**^
2. Number of missed days (necessity of bed rest or isolation due to migraine)/month	11.75 ± 8.82	10.00	4.12 ± 4.70	2.00	0.001^**^
3. Number of limited days (patient's productivity was affected due to migraine)/month	9.81 ± 7.03	9.00	8.06 ± 7.69	5.00	0.426
4. Pain intensity [rating from 0 (no pain) to 10 (excruciating pain)]	8.69 ± 1.49	9.00	5.69 ± 1.78	6.00	0.001^**^
5. Number of acute medication days/month	17.38 ± 7.61	15.00	10.56 ± 9.03	7.50	<0.001^**^
6. Number of migraine attacks associated with accompanying symptoms (e.g., nausea, vomiting, aura, photophobia/photosensitivity, phonophobia/phonosensitivity, intolerance to smells, pain worsens with movement)/month (*n* = 14)	12.06 ± 8.63	12.50	6.38 ± 7.59	5.50	0.003^**^
7. Number of migraine attacks that were free from accompanying symptoms/month	5.86 ± 7.66	2.00	9.14 ± 10.25	5.00	0.138
8. Number of pain-free days/month	7.38 ± 5.62	7.00	15.75 ± 7.56	15.00	<0.001^**^

** *Significant difference at 1% level of significance*.

*For each migraine parameter, the mean (SD) and median were calculated. All migraine parameters improved significantly (p < 0.01) except for “number of limited days per month” and “number of migraine attacks free of accompanying symptoms per month” (p > 0.05)*.

The duration of the therapeutic effect of erenumab was found to be 21 days for 56.3% of the patients, while 37.5% of the participants reported being satisfied with a once-monthly injection. One person experienced no change in migraine parameters after erenumab treatment.

Conversion of medication-overuse headache was seen in 57.1% of patients (eight out of the 14 patients who fulfilled the criteria of MOH at the onset of trial). Five of these patients continued to use triptans throughout erenumab therapy, however at a lower frequency. Additionally, reversion of chronic migraine to episodic migraine was noticed in nine (56.3%) patients in the study, i.e., nine patients had <15 migraine days per month by the end of erenumab treatment.

In addition to the migraine parameters, it was noted that in two patients the number of concurrent preventative treatments used at the onset of the trial decreased. Specifically, one patient who was using TCAS and ARBs at the onset of the study was able to stop them but had to be prescribed an SSRI (venlafaxine) due to concurrent anxiety during his/her treatment. Another patient who was prescribed erenumab as second line to anti-epileptics was able to continue only with erenumab. Moreover, while nine patients (56.3%) continued to use triptans at the initiation of the erenumab therapy, one patient, who was prescribed erenumab as a third line of treatment, reported stopping completely the use of triptans during erenumab treatment.

#### Safety and Tolerability

Treatment-related adverse effects were consistent with previous experiences with erenumab therapy. Overall, six patients reported no side effects, four mentioned flu-like symptoms following erenumab injection that lasted for 2–3 days, another four reported constipation, two mentioned dizziness on the day of the injection, one reported suffering from insomnia, and another one mentioned having migraine after erenumab injection. No serious adverse effects were seen. On the whole, erenumab proved to be a well-tolerated treatment, and no patient discontinued due to side effects.

## Discussion

The Republic of Cyprus has a population of 875,899 (2019) and gross domestic product per capita of 27,858 USD (2019 World Bank data), ranking 35th among world economies. Furthermore, it has the highest percentage (58.8%) of citizens of working age (30–34-year-olds) who have higher-level education in the European Union (24). This reflects the high socio-economic status as well as the relatively high purchasing power of its workforce, which allows a small proportion of patients to purchase erenumab out of the pocket despite its high cost. However, although Cyprus, as a nation, carries a similar burden of migraine as that of other developed countries, the fact that the percentage of chronic migraine sufferers in any given population set is small and that all other preventative medications are available for free or at a much lower cost, a novel therapy like erenumab becomes the last choice of treatment not only from the part of the patient but often due to a lack of experience from the part of the neurologist as well. As a result, despite the high purchasing power of its workforce, only a small percentage of Cypriots end up trying erenumab, namely, the small proportion of migraineurs who suffer from chronic migraine and have already tried and failed several preventative treatments. Moreover, the Cypriot healthcare system allows for the uncontrolled purchase of over-the-counter codeine-containing and other headache relief preparations, thus exacerbating the problem of chronic migraines.

Our study is the first real-life study assessing the efficacy and the impact of erenumab as a prophylactic treatment for migraine on the HR-QoL of therapy-resistant patients in Cyprus. Overall, our clinical data suggest that, over a period of 6 months, erenumab was safe, well-tolerated, and effective in preventing migraine symptoms and improving QoL. This is consistent with the results of randomized clinical trials for both episodic and chronic migraine patients with previous treatment failures (11, 25–28).

Most migraine parameters compared before and after erenumab treatment showed a significant improvement (*p* ≤ 0.001). MMDs were found to decrease from an average of 22 to an average of 13, while the number of missed days per month dropped from 11.75 to 4.12, and the pain-free days almost doubled (Table 3). Furthermore, conversion of medication-overuse headache was observed in 57.1% of patients, and reversion of chronic to episodic migraine was seen in 56.3% of patients. A *post-hoc* analysis of a randomized, double-blind study showed that erenumab treatment caused a decrease in migraine frequency large enough to reverse more than 50% of patients from chronic to episodic migraine (29). However, it is worth noting that the significant decrease observed in the MMDs and the number of missed days in our study was not seen in the number of limited days (i.e., days with decreased productivity due to migraine) which remained almost unchanged after erenumab therapy. This provides evidence that erenumab induces not only a quantitative effect on migraine parameters but also a qualitative one by allowing the patients to perceive their migraine attacks as less severe.

Even though several recent real-life studies have further confirmed the efficacy and tolerability of erenumab in medication-refractory patients (9–11, 13, 15), only a few have investigated directly the impact of erenumab on the aspects of migraine burden such as the QoL or the levels of anxiety and depression in chronic migraine sufferers (10, 13, 30). Consistent with previous findings (13), 81.3% of the patients in our study reported an improvement of their mental well-being and anxiety and depression levels, all in the first 3 months of treatment (Figure 1). This high percentage once again reflects the qualitative, not just quantitative, add-on value of erenumab treatment. A study by Russo et al. demonstrated a statistically significant reduction of migraine impact on everyday activities (using validated questionnaires) from the first month of erenumab therapy (10). Our data revealed good HR-QoL as assessed by the MSQ V2.1 in the last month of individual treatment for each patient. As seen in Table 2, the mean values of all three domains of the questionnaire were higher than 60 (on a scale from 0 to 100), demonstrating good overall daily functioning, with the RP domain having the highest health score (85). RP assesses the extent to which daily activities are completely interrupted; thus, its high value might be an indication that erenumab is highly efficacious in improving the ability of the patient to complete the daily activities, thus being able to return to the workforce, reducing missed workdays, and increasing productivity. Note is made of the fact that the assessment of 5-year long-term therapy of erenumab by a randomized clinical trial demonstrated not only sustainable reductions in migraine frequency but also a sustainable improvement of HR-QoL (30).

Moreover, subjective patient assessment at the 3-month interim point showed that erenumab had a positive effect on migraine frequency, pain intensity, and AMDs. In fact, as early as 3 months into the study period, 75% of the study patients reported a perceived decrease in the use of acute medication, and more than 80% of the patients reported an improvement of all assessed migraine parameters, except for a decrease in migraine duration which was still reported by 68% of the patients (Figure 1). In a similar manner, using data from the phase 3b LIBERTY study (which confirmed the efficacy and safety of erenumab in episodic migraine patients with two to four preventative treatment failures), Lanteri-Minet et al. analyzed the effect of erenumab on patient-reported outcomes in order to determine the impact of erenumab on impaired functioning and work-related disability caused by migraine symptoms, and they concluded that the augmentation of everyday activities and work productivity translated into improvement of the QoL of patients (28).

The safety and tolerability profile of erenumab in our study was comparable to that of available clinical trials. The two most common side effects were constipation (25%) and flu-like symptoms (25%), and no patient discontinued treatment due to adverse effects.

Our study had some limitations. Firstly, the sample size was small, which can be explained by several factors: the short recruitment period of the study, the lack of specialized headache centers on the island leading to hesitancy among neurologists to prescribe a novel agent such as erenumab, and the significantly lower cost of existing alternative prophylactic medications for chronic migraine. However, despite the small sample size, a number of the migraine parameters followed a normal statistical distribution, and thus parametric tests could be used for comparisons, while the other migraine parameters also provided valid and statistically significant results. Note that our results show that most of the patients are employed and educated, thus belonging to a group of high socioeconomic status. As such, the results cannot be representative for the whole population of migraine sufferers in Cyprus. Moreover, the fact that the erenumab response in our study was in line with larger, already-published clinical trials further confirms its efficacy and tolerability. Secondly, it is possible that the high cost of the monthly administered injection and the fact that most patients had to pay out of their pocket had created a placebo-like effect on the patients, leading to an exaggeration of the already positive effect of the medication. Arguably though, this somewhat-amplified patient-reported outcome could be the result of the qualitative effect of erenumab on everyday functioning. The highly significant results in the statistical tests with small probability values may additionally lead to the conclusion that the improvement in migraine parameters was not due to chance or psychological (placebo-type) confounding. Lastly, like in most real-life studies, the effect of concurrent oral preventative treatments could not be assessed due to heterogeneity. Nonetheless, despite treatment heterogeneity, there seems to be a uniform response both in terms of the efficacy of erenumab treatment as well as on HR-QoL parameters, satisfying both the primary and secondary endpoints of the study.

## Conclusion

Larger-scale trials on the effect of erenumab on the quality of life of patients with migraine will need to be conducted in order to validate our findings. However, these suggest that this novel mAB targeting the CGRP receptor may be a cost-effective way of reducing the burden of chronic migraine not only by improving their objective migraine parameters but also by subjectively improving their quality of life. This is a strong cost–benefit argument for the inclusion of erenumab in the Cypriot or any other country's healthcare formulary.

## Data Availability Statement

The original contributions generated for the study are included in the article/Supplementary Material, further inquiries can be directed to the corresponding author/s.

## Ethics Statement

The studies involving human participants were reviewed and approved by Cyprus National Bioethics Committee (CNBC). The patients/participants provided their written informed consent to participate in this study.

## Author Contributions

AT contributed to data collection and manuscript preparation. HT contributed to statistical analysis. CM contributed to the design of the study, critical review of manuscript drafts, and editing. All the authors contributed to the article and approved the submitted version.

## Conflict of Interest

The authors declare that the research was conducted in the absence of any commercial or financial relationships that could be construed as a potential conflict of interest.

## Publisher's Note

All claims expressed in this article are solely those of the authors and do not necessarily represent those of their affiliated organizations, or those of the publisher, the editors and the reviewers. Any product that may be evaluated in this article, or claim that may be made by its manufacturer, is not guaranteed or endorsed by the publisher.

## Footnotes

^1^Available online at: https://eprovide.mapi-trust.org/instruments/migraine-specific-quality-of-life-questionnaire.
