# Erratum: Assessment of the Effect of Erenumab on Efficacy and Quality-of-Life Parameters in a Cohort of Migraine Patients With Treatment Failure in Cyprus

**DOI:** 10.3389/fneur.2021.793620

**Published:** 2021-11-03

**Authors:** Frontiers Production Office

**Affiliations:** Frontiers Media SA, Lausanne, Switzerland

**Keywords:** erenumab, chronic migraine, quality of life, MSQ V2.1, Cyprus, treatment failure

Due to a production error, there was an error in affiliation 1 for author Andria Tziakouri. Instead of “Department of Neurology, Medical School, University of Nicosia, Nicosia, Cyprus,” it should be “Department of Neurology, American Medical Center, Nicosia, Cyprus.”

The publisher apologizes for this mistake. The original version of this article has been updated.

