# Supplementary material for: Assessment of the Effect of Erenumab on Efficacy and Quality-of-Life Parameters in a Cohort of Migraine Patients With Treatment Failure in Cyprus
Source: Front Neurol. 2021 Jul 29;12:687697. doi: 10.3389/fneur.2021.687697 (PMC8358110; doi:10.3389/fneur.2021.687697)
Supplement: Supplementary file 1 [file Image_1.pdf]

Appendix A

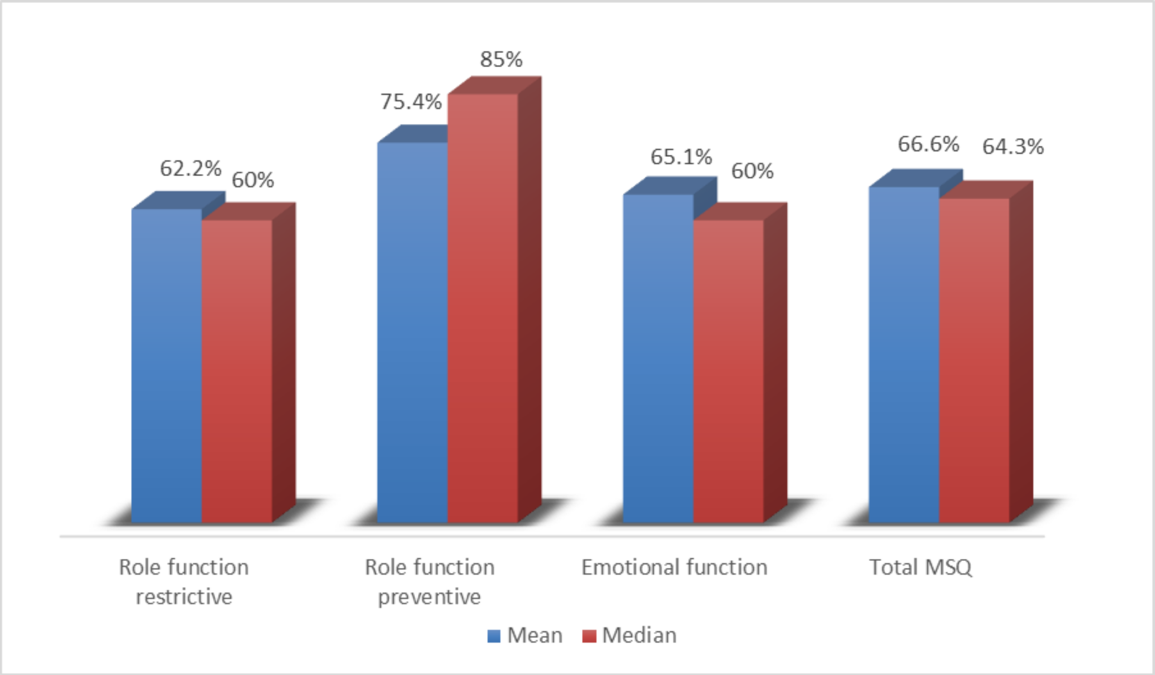

Figure 2: Descriptive statistics for the Migraine-specific-quality of life questionnaire (MSQ V2.1). Mean/Median of transformed domain scores (scale 0-100), assessed during the last four weeks of erenumab treatment.
