# Supplementary material for: Assessment of the Effect of Erenumab on Efficacy and Quality-of-Life Parameters in a Cohort of Migraine Patients With Treatment Failure in Cyprus
Source: Front Neurol. 2021 Jul 29;12:687697. doi: 10.3389/fneur.2021.687697 (PMC8358110; doi:10.3389/fneur.2021.687697)
Supplement: Supplementary file 2 [file Image_2.pdf]

Appendix B

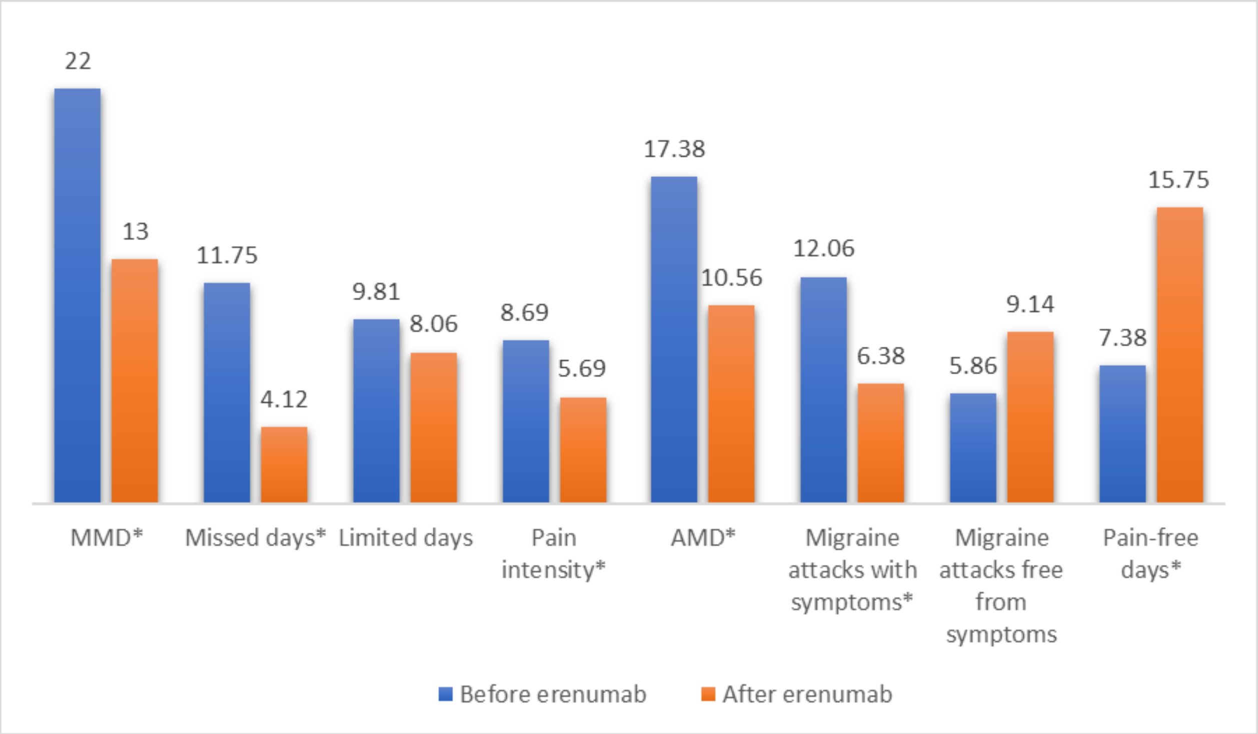

** Difference is significant (p<1%)*

Figure 3: Migraine parameters before and after erenumab therapy (mean values). All migraine parameters improved significantly ($p<0.01$), except for "number of limited days per month" and "number of migraine attacks free of accompanying symptoms per month" ($p>0.05$).
